# Calcium-Modification Techniques in Calcified Coronary Artery Disease: A Systematic Review of Comparative Evidence on Procedural Success, Complications and Clinical Outcomes

**DOI:** 10.3390/jcm15218319

**Published:** 2026-10-26

**Authors:** Sundar Chidambaram, Manokar Panchanatham, Mahadevan Venkataramani, Maheshbabu Panchanatham, Jebaraj Rathinasamy, Deepchand Raja, Nithesh Kumar, Surendar Gunasekaran, Selva Prakash, Nagendra Boopathy Senguttuvan, Vaikkom S. Mahadevan, Ashish H. Shah

**Affiliations:** 1 Department of Cardiology, Kauvery Hospital, Vadapalani, Chennai 600026, India; pmanokar@hotmail.com (M.P.); daffodils.amm@gmail.com (M.V.); maheshgaja@gmail.com (M.P.); jebaraj66@gmail.com (J.R.); deepchandh@gmail.com (D.R.); mail2drnithesh@gmail.com (N.K.); suren.cardiocare@gmail.com (S.G.); selvaaa002@gmail.com (S.P.); 2 Department of Cardiology, Sri Ramachandra Institute of Higher Education and Research, Chennai 600116, India; drsnboopathy@gmail.com; 3 Cardiology, Chan School of Medicine, University of Massachusetts, Worcester, MA 01655, USA; vs.mahadevan@gmail.com; 4 Cardiology, St. Boniface Hospital, University of Manitoba, 409 Taché Avenue, Winnipeg, MB R2H 2A6, Canada; ashish.shah@umanitoba.ca

**Keywords:** coronary artery calcification, percutaneous coronary intervention, rotational atherectomy, orbital atherectomy, intravascular lithotripsy, calcium modification, intravascular imaging, systematic review

## Abstract

**Background/Objectives:** Severe coronary calcification complicates percutaneous coronary intervention (PCI), and several calcium-modification technologies are available, but direct randomized comparisons are limited. We reviewed the comparative evidence on procedural success, complications and clinical outcomes. **Methods:** Comparative studies of coronary calcium-modification techniques during PCI that reported procedural, safety or clinical outcomes were eligible. PubMed and ClinicalTrials.gov were searched in May 2025 for records published from 1 April 2015 to 1 April 2025 (PROSPERO CRD420251077471; no external funding). We included 32 publications: eight randomized trials (10 publications) and 22 observational or descriptive studies. Evidence was organized in tiers and synthesized descriptively, without pooling, because definitions were heterogeneous and event rates were low. Risk of bias in randomized trials was assessed with RoB 2 where full texts were accessible. **Results:** Procedural success was generally high (usually 85–100%) and major complications were uncommon, but success definitions differed, and reported dissection (0% to over 80%) and slow-flow (0% to 31.6%) rates varied widely. Most comparative data were observational. In randomized comparisons, no device consistently outperformed another for procedural success, stent expansion or minimal stent area. Clinical outcome comparisons were limited by heterogeneous endpoints and low event rates. Risk of bias was rated from full texts for five out of the eight randomized trials, none at low risk overall; ratings for the others were provisional or not possible. **Conclusions:** The evidence is largely observational and of low certainty, and is insufficient to establish clinically meaningful differences among most technologies. Device selection should be lesion-specific and imaging-informed, and adequately powered head-to-head trials with standardized endpoints are needed.

## 1. Introduction

Coronary artery calcification (CAC) is increasingly common in patients undergoing percutaneous coronary intervention (PCI), affecting nearly one-quarter to one-third of cases [1,2]. Its prevalence continues to rise with aging and comorbidities such as diabetes and chronic kidney disease [3]. CAC reflects advanced atherosclerosis and is associated with reduced procedural success and higher rates of complications, target lesion failure, and major adverse cardiovascular events (MACEs) compared to non-calcified lesions [2].

Severe calcification impedes balloon and stent delivery, contributes to under-expansion and malapposition, and increases the risk of perforation, dissection, acute vessel closure, and stent thrombosis and long-term stent failure [4,5,6,7]. Although these complications may occasionally require emergency surgery, the vast majority are now managed successfully with contemporary percutaneous techniques such as covered stents, prolonged balloon tamponade, or vasodilator therapy. Stent under-expansion is a major predictor of adverse outcomes [5]. Consequently, adequate calcium modification has become essential for successful PCI.

Multiple devices exist for calcium modification. Rotational atherectomy (RA) uses a diamond-coated burr rotating at high speeds to ablate superficial calcium and improve vessel compliance [8]. Orbital atherectomy (OA) employs an eccentrically mounted, orbiting crown that sands plaque bidirectionally, allowing treatment of both concentric and eccentric calcium [8,9]. Intravascular lithotripsy (IVL) delivers circumferential shockwaves to fracture deep and superficial calcium with low inflation pressures, reducing vascular trauma while improving stent expansion [3,7]. Cutting and scoring balloons create controlled intimal fractures through microblades or scoring elements, enabling lesion preparation at lower pressures [3]. Excimer laser coronary angioplasty (ELCA) ablates plaque via photochemical and photothermal mechanisms but is less effective in heavy calcification and is generally reserved for undilatable lesions or in-stent restenosis [8].

These technologies act through fundamentally different mechanisms: RA, OA and ELCA have an ablative component, IVL primarily fractures calcium, and cutting/scoring balloons modify vessel compliance without true plaque debulking. We therefore use the terms “calcium modification” and “lesion preparation” throughout, rather than “debulking”, which would obscure these differences.

Despite the expanding evidence base, many earlier syntheses addressed pairwise questions or a single technology versus heterogeneous alternatives [10,11,12,13,14]. Two recent network meta-analyses of randomized trials have since placed a broader range of strategies within one framework: Maremmani et al. compared ELCA, IVL, modified balloons, OA, RA and RA plus cutting balloon with a conventional strategy [15], and Simonetti et al. evaluated eight calcium-modification strategies across imaging, angiographic, procedural and clinical outcomes [16]. We do not seek to replicate these analyses. The contribution of the present work is different: (i) a systematic review that keeps randomized head-to-head evidence, randomized comparisons against balloon-based preparation, and observational comparative evidence as separate evidence tiers; (ii) a descriptive synthesis of procedural success, complications, short- to medium-term clinical outcomes and imaging findings, including data on OA and ELCA; and (iii) an explicit account of the limits of the available evidence.

Accordingly, we conducted a systematic review of contemporary coronary calcium-modification strategies. Our objective was to describe and compare, without quantitative pooling, procedural success and safety while explicitly defining the limits of the evidence and identifying areas in which device selection must still rely on lesion characteristics, imaging findings, operator expertise, and non-randomized data.

## 2. Materials and Methods

### 2.1. Study Design and Registration

This systematic review was conducted and reported in accordance with the Preferred Reporting Items for Systematic Reviews and Meta-Analyses (PRISMA) 2020 statement [17]. The completed PRISMA 2020 checklist and PRISMA 2020 for Abstracts checklist are provided in Supplementary File S1. The review was registered with the International Prospective Register of Systematic Reviews (PROSPERO) (registration number CRD420251077471; registered on 25 June 2025). The registration record and the full protocol are publicly available at https://www.crd.york.ac.uk/PROSPERO/view/CRD420251077471 (accessed on 11 October 2026).

Amendments to the registered protocol. The review was registered after the searches and screening had been completed and before data extraction began. The registered protocol planned random-effects pairwise meta-analyses and a frequentist network meta-analysis. The review differs from the registered protocol as follows: (i) no quantitative pooling, including network meta-analysis, was performed, and results were synthesized descriptively for the reasons given in Section 2.6; (ii) the protocol listed CENTRAL, Embase, MEDLINE, PubMed and Scopus, but only PubMed and ClinicalTrials.gov were searched, supplemented by reference lists and conference proceedings (Section 2.3); (iii) study authors were not contacted for missing or unclear data; (iv) the protocol excluded single-arm studies, but a limited number of single-arm cohorts and their subgroup analyses were retained for descriptive context only and were not used for comparative statements (Section 2.4); (v) procedural success was recorded as defined by each study rather than by the protocol definition (residual stenosis < 30% with TIMI 3 flow and no major complications), because definitions varied across studies (Section 2.5); (vi) the protocol planned risk-of-bias assessment with RoB 2 and the Newcastle–Ottawa Scale, assessment of reporting bias with funnel plots and Egger’s test, and GRADE assessment of certainty, whereas only RoB 2 was applied, and only to the randomized trials (Section 2.7); and (vii) the additional outcomes of fluoroscopy time, contrast volume, length of hospital stay, bailout device use and device-related technical failure were not synthesized.

### 2.2. Research Objectives

The primary objective of this systematic review was to describe and, where possible, compare procedural success among contemporary coronary calcium-modification techniques used during PCI, including RA, OA, IVL, cutting and scoring balloons, and ELCA. Procedural success was selected as the primary outcome because it was consistently reported across the majority of included studies and represents the immediate clinical objective of calcium modification during PCI. We acknowledge that this outcome is a surrogate whose definition varies between studies (Section 4.4).

Secondary objectives were to describe periprocedural complications (including coronary perforation, severe dissection, and slow or no reflow) and short- to medium-term clinical outcomes, including target lesion revascularization (TLR), MACEs, and all-cause mortality. Exploratory analyses assessed whether lesion characteristics or patient subgroups influenced device performance; these were descriptive and based on subgroup results reported by individual studies.

### 2.3. Search Strategy and Data Sources

PubMed and ClinicalTrials.gov were searched for records published between 1 April 2015 and 1 April 2025. The PubMed query was restricted to English-language human studies and combined MeSH terms and free-text keywords for coronary artery disease or coronary artery calcification with each calcium-modification modality (rotational atherectomy, orbital atherectomy, intravascular lithotripsy, cutting balloon, scoring balloon and laser atherectomy), limited to the publication types Comparative Study and Randomized Controlled Trial. The complete PubMed search string is given in Supplementary Table S1; ClinicalTrials.gov was searched for registered studies on the same calcium-modification modalities. The searches were run in May 2025.

**Figure 1:**
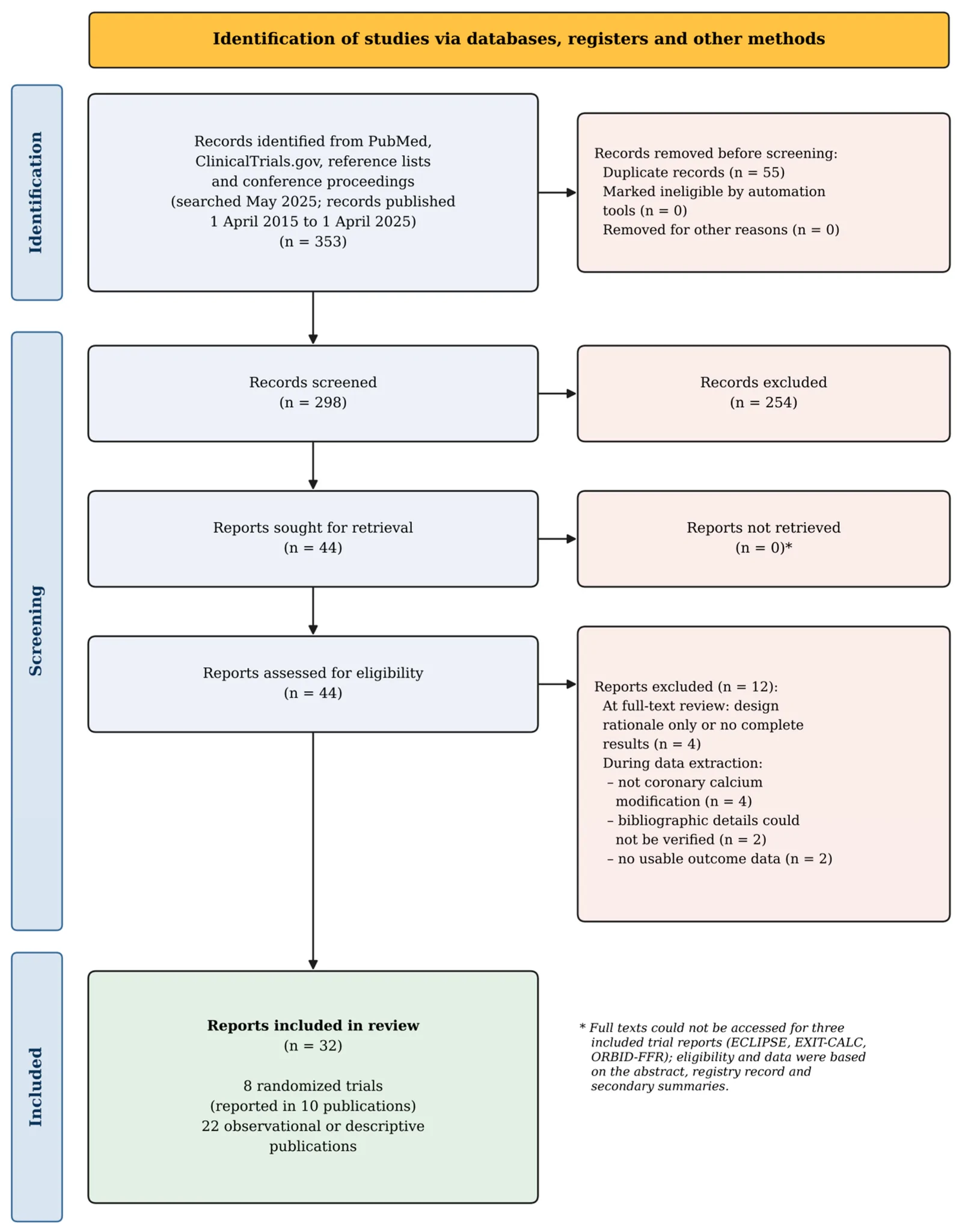
PRISMA 2020 flow diagram of study selection. Twelve out of the 44 reports assessed for eligibility were excluded: 4 at full-text review (design rationale only or no complete results); 4 found during data extraction not to evaluate coronary calcium modification (references [18,19,20,21]); 2 whose bibliographic details could not be verified at the time of the eligibility audit [22,23]; and 2 without usable outcome data (Yasumura et al. [24] and the STIFF study [25]). Randomized trials published after the search date (Section 3.7) are not included.

To enhance completeness, additional manual searches of reference lists from relevant reviews and key trials, as well as major cardiology conference proceedings, were conducted to identify eligible unpublished or ongoing comparative studies. Records identified through these supplementary searches are included in the total shown in Figure 1. Duplicate records were removed before screening, and no automation tools were used at any stage. Searching a single bibliographic database and filtering by publication type are limitations that we discuss in Section 4.4.

Randomized trials reported after the search date (BALI, ShortCUT and VICTORY, and two recent network meta-analyses) were identified through targeted, non-systematic searching in April 2026 and are described narratively in Section 3.7. They are not part of the 32 included publications.

### 2.4. Study Selection

Study selection was performed in a two-stage process. First, titles and abstracts were screened to exclude clearly irrelevant studies. Second, full-text articles were reviewed to determine final eligibility based on predefined inclusion and exclusion criteria. At both stages, three reviewers independently assessed each record or report, and disagreements were resolved by discussion and consensus.

Studies were included if they evaluated at least one coronary calcium-modification technique during PCI and reported outcomes related to procedural success, complications, or follow-up clinical events. The primary evidence base comprised comparative studies (randomized controlled trials and comparative observational or registry studies). In addition, a limited number of single-arm device studies and subgroup analyses of single-arm device cohorts (for example, ORBIT II sub-analyses) were retained for contextual, descriptive information on device-specific outcomes in patient subgroups (Section 3.6). These non-comparative studies were not used for any comparative statement on effectiveness or safety. Studies were excluded if they were case reports, small case series, narrative reviews, editorials, non-coronary studies, or if they lacked sufficient outcome data. When multiple publications reported overlapping patient populations, the most comprehensive or recent dataset was used, and publications arising from the same trial or cohort (for example, the ROTA.shock main trial and its sub-studies, and multiple ORBIT II sub-analyses) were counted as one population in study counts.

The study-selection process is shown in Figure 1. The searches identified 353 records; after removal of 55 duplicates, 298 records were screened by title and abstract and 254 were excluded. Forty-four reports were sought and assessed for eligibility, and four were excluded at full-text review (design rationale only or no complete results), leaving 40 studies for data extraction. During extraction, four further reports were found not to evaluate coronary calcium modification (a cohort comparing chronic-total-occlusion recanalization with complex non-occlusive PCI [19], a randomized trial of scoring versus conventional balloon predilation that enrolled non-calcified lesions [20], a trial of a drug-coated scoring balloon for in-stent restenosis [21], and a decision-analytic cost-effectiveness model [18]) and were removed, leaving 36 publications. Two further publications (an ORBIT II left-main subgroup analysis [22] and a retrospective analysis of heparin versus bivalirudin during orbital atherectomy [23]) were then removed because their published reference details could not be verified at the time of the eligibility audit, leaving 34 publications. Two of these (Yasumura et al. [24] and the STIFF study [25]) had no usable outcome data and were also removed, leaving 32 publications.

In total, 12 out of the 44 reports assessed for eligibility were excluded: the four excluded at full-text review were design-rationale papers or reports without complete results, and the eight excluded during data extraction are identified above and listed with reasons in Supplementary Table S2. Full texts could not be accessed for three included trial reports (ECLIPSE, EXIT-CALC and ORBID-FFR); for these, eligibility and data were based on the abstract, the registry record and secondary summaries (Section 2.7).

To avoid misinterpretation, observational studies were not described using randomized trial terminology (e.g., “trial” or “arm”). Data extraction was performed independently by two reviewers using a standardized data extraction form. Extracted variables included study design and sample size; unit of analysis (patient or lesion); patient demographics and comorbidities; lesion characteristics and definitions of coronary calcification severity; calcium-modification techniques and adjunctive intravascular imaging guidance; procedural outcomes, including procedural success and its definition in each study; periprocedural complications (coronary dissection, perforation, and slow or no reflow); and clinical outcomes, including TLR, MACEs as defined by individual studies, and all-cause mortality, assessed during in-hospital stay and available short- to medium-term follow-up. Discrepancies were resolved by consensus or by consultation with a third reviewer. Study investigators were not contacted to obtain or confirm data; information that was missing or unclear in the published reports was recorded as not reported (n/r), and no assumptions were made about missing values. Definitions of MACEs varied across studies and typically included combinations of death, myocardial infarction, and repeat revascularization; MACE comparisons therefore reflect study-specific definitions rather than a harmonized endpoint. Coronary dissections were classified according to the National Heart, Lung, and Blood Institute (NHLBI) classification, with severe dissections defined as Types D–F.

### 2.5. Outcomes of Interest

The primary outcome was study-defined procedural success, as defined by individual studies (typically successful lesion preparation and stent deployment with acceptable angiographic results). Secondary outcomes included coronary perforation, dissection, slow or no reflow, TLR, MACEs, all-cause mortality and intravascular imaging findings. The definition of procedural success used by each included study is recorded in Supplementary Table S3.

### 2.6. Data Synthesis

Studies were synthesized descriptively, by evidence tier. Outcomes are reported as given in each study (percentages or counts per arm, with the study-specific definition). No pooled effect estimates were calculated and no network meta-analysis was performed; because only two independent randomized trials (ROTA.shock and ROLLER COASTR-EPIC22) were connected by a common comparator, there was no closed loop; procedural-success and MACE definitions differed between studies; and event rates were low. No equivalence or non-inferiority margin was applied, and the absence of a reported difference between devices is not interpreted as evidence that they are equivalent.

Grouping for synthesis. Studies were grouped into three evidence tiers: (1) randomized device-versus-device comparisons; (2) randomized comparisons against conventional balloon-based preparation; and (3) observational comparative studies. Single-arm cohorts and subgroup analyses of single-arm cohorts were described separately (Section 3.6) and were not used for comparative statements. Within each tier, studies were grouped by the devices compared and by outcome. This grouping was not prespecified in the registered protocol.

Effect measures. No summary effect measure was calculated. Dichotomous outcomes are presented as proportions or counts per group, and continuous imaging outcomes as means (±SDs) or medians per group, as reported. Between-group effect estimates (for example, risk ratios or mean differences), confidence intervals and *p*-values are presented only where they were reported by the original study; no data were converted, recalculated or imputed.

Presentation of results and exploration of heterogeneity. Results are tabulated by evidence tier and outcome in the tables in Section 3, with study-level data for all included publications in Supplementary Table S3. Because no pooled estimates were produced, no statistical measures of heterogeneity were calculated and no meta-regression, subgroup meta-analysis or sensitivity analysis was performed. Possible sources of variation between studies (design, outcome definitions, unit of analysis, follow-up duration and lesion characteristics) were examined descriptively. The decision not to pool followed Cochrane guidance on situations in which meta-analysis is inappropriate [26].

The unit of analysis (patients, lesions or imaging segments) is stated for each study in Supplementary Table S3. Publications arising from the same trial or cohort (for example, the ROTA.shock main report and its sub-studies, and the ORBIT II sub-analyses) were treated as one population and are not counted as independent evidence. Anatomical complexity (for example, ostial, left main, long, bifurcation and balloon-uncrossable lesions) and calcium morphology are plausible effect modifiers that were not consistently reported, so differences between studies in lesion mix limit any cross-study comparison (Section 4.2).

### 2.7. Risk of Bias, Reporting Bias and Certainty of Evidence

Risk of bias in the randomized trials was assessed with the Cochrane RoB 2 tool for the effect of assignment to the intervention (intention to treat), across five domains: the randomization process, deviations from intended interventions, missing outcome data, measurement of the outcome and selection of the reported result. Each domain was judged as low risk, some concerns or high risk, and the overall judgment followed the least favorable domain. Judgments were based on the published reports and, where accessible, registry records and supplements. A domain was labeled “not assessable” when the text did not allow a judgment, and a trial was labeled “not assessed” when its full text could not be accessed; no rating was inferred in either case. Observational studies were not rated with ROBINS-I. Instead, the design features most relevant to bias (open-label allocation, single-center setting, retrospective design, unit of analysis and overlap between publications) were recorded in Supplementary Table S2, and these studies were regarded as being at risk of confounding and selection bias by design. The certainty of evidence was not graded with GRADE or CINeMA, and no certainty rating is claimed; evidence from observational and small single-center randomized studies was regarded as being of low certainty on this basis. This limitation is discussed in Section 4.4.

Risk-of-bias judgments were made independently by three reviewers, and disagreements were resolved by discussion and consensus. Risk of bias due to missing results (reporting bias) was not formally assessed: with no pooled analysis and few studies per comparison, funnel plots and statistical tests for small-study effects were not applicable. Selective reporting within the randomized trials was considered in RoB 2 domain 5, and ClinicalTrials.gov was searched to identify registered trials without published results.

## 3. Results

### 3.1. Study Characteristics

A total of 32 publications (2016 to 2025) on calcified coronary artery disease interventions were included after the eligibility audit (Figure 1) [27,28,29,30,31,32,33,34,35,36,37,38,39,40,41,42,43,44,45,46,47,48,49,50,51,52,53,54,55,56,57,58]. They comprised eight distinct randomized trials, reported in 10 publications (ROTA.shock accounts for three), and 22 observational or descriptive publications, among them several sub-analyses of the ORBIT II cohort. Sample sizes ranged from about 20–80 patients in small OCT imaging sub-studies to about 1000 per arm in large pragmatic trials, and real-world registries were also included. Patients were typically elderly (mean age: about 70–80 years) and had a high burden of hypertension, diabetes and multivessel disease. Lesions were severely calcified, as defined by angiography or intravascular imaging; several studies also stratified by lesion complexity (for example, chronic total occlusion versus non-occlusive lesions). Techniques studied included RA, OA, IVL, cutting/scoring balloons and ELCA, alone or in comparison. Many studies were head-to-head comparisons; others were single-arm device cohorts or subgroup analyses of such cohorts, which are described separately (Section 3.6) and were not used for comparative inference.

Evidence was organized in three tiers: (1) randomized device-versus-device comparisons; (2) randomized comparisons against conventional balloon-based preparation (for example, ECLIPSE); and (3) observational comparative studies. These tiers carry different evidentiary weights and are not pooled conceptually. The unit of analysis also differed between studies (patients, lesions, or imaging segments); for example, ECLIPSE randomized 2005 patients who had 2492 lesions. Because multiple lesions within one patient are not independent, the unit of analysis is stated in Table 1.

### 3.2. Procedural Success

Procedural success was generally high, with all calcium-modification strategies in the studies reporting it, but the definition of success differed between studies, so absolute rates are not directly comparable. In one large RCT of OA vs. modified balloon angioplasty, success rates were 85.5% vs. 86.1% (strictly defined as achieving post-stent luminal criteria) [27]. Most smaller studies and registries reported success rates of 95–100% for RA, IVL or OA. In RA–IVL comparisons, success was 100% vs. 94% in the randomized ROTA.shock trial [28] and 95.8% vs. 99.3% in a retrospective single-center comparison of RA and IVL [38]. A model-based economic evaluation of OA versus RA in Japan [18] was not treated as clinical evidence, and its inputs were not used. In general, there were no statistically significant differences between techniques in the studies that tested them. Other side-by-side comparisons of RA with IVL or OA [30,31,32,33,34,35] were retrospective analyses or sub-analyses of the same randomized population (ROTA.shock) and reported similarly high success rates; the RA-versus-OA comparison [34] was a retrospective study, not a randomized comparison. Only the large OA-versus-balloon RCT reported lower success (about 85%), probably because of its more stringent endpoint; even there, the difference between arms was not significant [27]. Residual-stenosis thresholds, requirements for no alternative device, and imaging criteria all varied, so success may not measure the same construct across studies, and the high rates in many studies leave little room for differences to appear (a ceiling effect). Table 1 summarizes procedural outcomes for all randomized trials and for comparative observational studies with at least 100 patients; outcome data for all 32 publications are given in Supplementary Table S3.

### 3.3. Procedural Complications

Across the included studies, overall complication rates were low for all calcium-modification techniques, and no single modality demonstrated a consistent safety advantage in head-to-head comparisons; however, low event rates and the predominance of observational data limit the power to detect differences. The most commonly reported periprocedural adverse events included coronary perforation, coronary dissection, and slow- or no-reflow phenomena.

Coronary perforation was uncommon in the studies that reported it. In the randomized trials, perforation ranged from 0% (ROTA.shock, ROTA-CUT, EXIT-CALC, the Zou trial and Kini) to 3.5% (the RA and ELCA arms of ROLLER COASTR-EPIC22) and was 2.7% in both arms of ISAR-CALC [28,29,36,37,50,51,58]; ECLIPSE described perforation as very infrequent without arm-specific figures [27]. In observational comparisons, perforation occurred in 0.7% with IVL versus 0.8% with RA [38], in 1.0% with IVL versus 2.1% with any atherectomy [31], and in 0–2% per group in RA–OA comparisons [33,34]. Perforation-related mortality was not extracted separately, so we make no statement about it.

Reported dissection rates depended strongly on the definition used and on whether intravascular imaging was applied. Angiographic dissection was infrequent in the comparative studies that reported it: 1.7% (RA), 7.0% (IVL) and 5.2% (ELCA) in ROLLER COASTR-EPIC22 [36]; 1.5% with IVL versus 1.2% with atherectomy in registry data [31]; and 0% with IVL versus 1.3% with RA [38]. Higher figures were reported when any dissection was counted or when OCT was used: 63.2% versus 81.0% after IVL versus conventional preparation (*p* = 0.056) in EXIT-CALC [50]; 77.8% after RA and 75.0% after IVL in the ROTA.shock OCT sub-study, none requiring bailout intervention [30]; 21.4% versus 44.8% after RA with a cutting balloon versus a non-compliant balloon in ROTA-CUT [37]; and 18.2% with a scoring balloon versus 0% with a cutting balloon after OA [54]. OCT-guided RA and IVUS-guided RA detected trailing-edge dissections in 3% and 5.9% of cases, respectively [43]. Across studies, therefore, reported dissection ranged from 0% to over 80%, and no single threshold applies; clinical consequences were rarely reported.

Slow flow or no reflow was reported in 0% to 31.6% of patients, depending on the study and definition. Most comparative studies reported 0–2% [27,31,36,38], with 1.4% after OA versus 0.4% after balloon angioplasty in ECLIPSE, a difference that was not statistically significant [27]. Higher rates were seen in a few studies: TIMI flow below 3 in 8.6% of the RA group and 13.9% of the cutting balloon group in the Kini side-branch trial [58]; 5% in both arms of the Zou trial [29]; 7% with RA versus 4% with OA in a retrospective comparison [34]; and six out of 19 patients (31.6%) in the step-up RA group versus none with Rota-Tripsy in a small retrospective study [53]. Many studies did not report this outcome.

Other complications were reported inconsistently. In-hospital myocardial infarction occurred in 2.9% of OA-treated and 6.4% of RA-treated patients in one comparison (not significant) [33]. The extracted data contained little information on stroke, tamponade or systemic embolism, and the absence of such reports should not be read as the absence of events. Procedural complication rates were in the low single digits in most studies, with the exceptions above. Given low event rates, heterogeneous definitions, and device selection driven by lesion anatomy, these data cannot exclude clinically relevant differences in safety between techniques.

### 3.4. Clinical Outcomes

Short- and medium-term outcomes, typically assessed at 6 to 12 months, were generally favorable across all modalities, and no technique demonstrated clear superiority in the studies that compared them. Reported endpoints included restenosis or TLR, target-vessel failure or MACEs (including cardiac death, myocardial infarction, or TLR), and all-cause mortality. TLR was 0–1 events per arm in most small comparative studies [28,29,37,38,50]; higher values were seen with a scoring balloon in ISAR-CALC (8.1% at 30 days) [51] and in ORBIT II sub-analyses (5–11% at one to three years; descriptive) [40,41,46]. In the 1-year results of the multicenter open-label RCT comparing orbital atherectomy with modified balloon angioplasty, target-vessel failure was 11.5% in the orbital atherectomy arm and 10.0% in the balloon angioplasty arm, a difference that did not reach statistical significance [27]. This trial’s finding is clinically informative: in lesions eligible for either strategy, routine OA did not improve the principal clinical or imaging outcomes compared with balloon-based preparation. Studies comparing IVL with RA reported minimal TLR, 0–1% in both groups at in-hospital to 6-month follow-up [28,38]. Target-vessel failure was approximately 10–11.5% in ECLIPSE (a composite including cardiac death, myocardial infarction and revascularization), and TLR was 0% after IVL or RA in the smaller comparative studies; these figures reflect different endpoints and populations and should not be compared directly.

MACE rates varied widely with definition and follow-up: 0% in several small studies, 10.3% at 30 days after RA with a cutting balloon in ROTA-CUT [37], 11.5% target-vessel failure at one year in ECLIPSE [27], and 27.8% at three years in the elderly subgroup of ORBIT II [40]. MACE definitions differed among studies: some reported cardiac death, MI and TLR, whereas others reported target-vessel failure or broader cardiovascular composites. The MACE data presented here therefore reflect study-specific definitions rather than a harmonized endpoint. In a retrospective comparison of RA and OA involving 117 patients, each arm recorded a 6% rate of MACEs at 30 days [34].

All-cause mortality was low in hospital and at follow-up; in-hospital or 30-day mortality was 0% in most small comparative studies but reached 1.5–2.7% in several larger series (2.7% with IVL and 2.2% with atherectomy in a registry [31]; 1.5% with RA [33]; 2% with OA [34]). At one-year, cardiac death occurred in 4.0% of patients treated with OA versus 2.7% of those treated with balloon angioplasty [27]. Smaller studies often observed 0–2% mortality per arm [33,34,38,39,44,45]. A summary of short- and medium-term clinical outcomes across key studies is provided in Table 2.

### 3.5. Technique-Specific Observations

RA is widely used as a first-line approach for heavily calcified lesions, with procedural success of 93% to 100% across the comparative studies reviewed. In a single-center retrospective cohort of orbital atherectomy, procedural success was 96.4% in diabetic and 100% in non-diabetic patients, with low acute complication rates (perforation 1.4% and slow flow 1.4% overall) [47]. A subgroup analysis of imaging guidance (OCT versus IVUS) in RA reported consistent outcomes with both modalities [43]. OA, predominantly studied in the United States, achieved procedural success ranging from 85% to 100%, with the largest RCT reporting success of 85.5% [27]. Smaller studies reported 100% success, and a subgroup analysis found no significant sex differences in outcomes [39]. In selected high-risk groups, such as patients with prior coronary artery bypass grafting, the incidence of in-hospital MACEs was elevated, but short- and medium-term outcomes were reported to align with those of lower-risk subgroups [48].

IVL achieved procedural success above 90% with few complications in the selected studies. A single-center randomized trial of IVL versus conventional PCI (20 patients per arm) [29] reported 100% procedural success with no perforation or dissection, and larger IVL–RA comparisons reported perforation in under 1% [38]. Short- and medium-term outcomes, including TLR and MACEs, were in the low single digits and similar to those seen with RA. Cutting and scoring balloons yielded technical success in 89–95% of cases [37,49]. Slightly lower acute success and higher dissection rates were reported with these balloons in some studies than with atherectomy, though bailout stenting rates remained low [37,49]; these cross-study observations are not a controlled comparison. Medium-term outcomes such as restenosis were not consistently reported for these devices [29,44].

The traditional assumption that cutting balloons are less effective in severe calcification rests mainly on early mechanistic studies and operator experience rather than robust randomized evidence. The ShortCUT randomized trial (413 patients at 21 US centers; IVUS-guided PCI), as presented at TCT 2025, found cutting balloon angioplasty to be non-inferior to IVL for post-PCI minimal stent area, with similar procedural success and safety [59]; subgroup results by planned atherectomy were reported separately and should be interpreted cautiously until the full peer-reviewed report is available. This suggests that cutting balloons may perform adequately in selected moderately to severely calcified, balloon-crossable lesions, but it should not be extrapolated to lesions in which atherectomy is most needed. However, most published studies included in this review did not stratify outcomes using standardized OCT/IVUS-based definitions of moderate versus severe calcium, precluding a reliable subgroup analysis. Future randomized trials incorporating imaging-defined calcium severity will be necessary to clarify differential device performance across the calcium spectrum.

ELCA has been evaluated in limited studies; in a three-arm RCT comparing RA, IVL, and ELCA, ELCA achieved procedural success of 89.5%, with perforation in 3.5% and MACEs in around 1.8% of cases, similar to RA and IVL, although with only 57 patients per arm the precision of these estimates is limited [36].

### 3.6. Subgroup Findings Reported by Individual Studies (Descriptive)

The following findings come from subgroup analyses within single studies, mostly of single-arm cohorts such as ORBIT II. They are hypothesis-generating, were not combined in any formal subgroup meta-analysis, and are not used for comparative inference. In patients with chronic kidney disease (CKD), an orbital atherectomy registry sub-analysis demonstrated that those with reduced estimated glomerular filtration rate (<90 mL/min) experienced a significantly higher rate of 30-day MACEs than patients with preserved renal function (12.6% vs. 3.7%), driven primarily by increased rates of myocardial infarction and target lesion revascularization; in-hospital mortality was also marginally higher (0.3% vs. 0%) [41]. In an ORBIT II sub-analysis, the one-year MACE rate was approximately 9–10% in both patients aged ≥75 years and younger patients [40].

Prior coronary artery bypass grafting (CABG) likewise portended worse outcomes: in one OA cohort, patients with a history of CABG had substantially higher in-hospital MACEs (16.9% vs. 6.4%) and increased one-year cardiac death rates (6.2% vs. 2.7%) compared to non-CABG patients, a disparity attributed to more complex lesion anatomy rather than differences in device performance [48]. Diabetes did not appear to modify the effectiveness of OA: a retrospective single-center study found similar procedural success and acute MACE rates in diabetic and non-diabetic patients undergoing OA [47]. Prior PCI status and sex did not significantly affect outcomes in the reported analyses; one analysis noted marginally higher procedural success in men than women (96.8% vs. 91.1%), without differences in complication or MACE rates [39]. A summary table comparing the advantages and disadvantages of each calcium-modification technique is presented in Table 3.

### 3.7. Randomized Evidence Published After the Search Date (Narrative)

Several randomized trials and two randomized-trial network meta-analyses became available after the search date. Because they were identified outside the systematic search, they are summarized narratively and are not part of the 32 included publications.

BALI randomized 200 patients at nine European centers to OCT-guided PCI with or without IVL added to conventional lesion preparation (rotational atherectomy was permitted by predefined criteria). The primary composite of procedural failure (failed stent delivery or residual area stenosis of 20% or more on OCT) or target-vessel failure at 1 year occurred in 35.4% with IVL versus 51.5% with conventional preparation (RR: 0.69, 95% CI: 0.48–0.97), a difference driven mainly by residual area stenosis rather than clinical events [60]. ShortCUT (Section 3.5) found cutting balloon angioplasty non-inferior to IVL for minimal stent area [59], and the VICTORY trial, as presented at TCT 2025, reported that a super-high-pressure non-compliant balloon was non-inferior to IVL for lesion preparation and stent expansion in severely calcified lesions [61]. ECLIPSE, already included, showed no significant reduction in target-vessel failure or increase in minimal stent area with routine OA compared with balloon angioplasty [27].

Two network meta-analyses restricted to randomized trials have also been published. Maremmani et al. reported that atherectomy-based strategies carried a higher risk of serious adverse events than a conventional strategy, driven by slow flow/no reflow, and that IVL offered the most favorable balance of safety and efficacy [15]. Simonetti et al. found that, compared with standard non-compliant balloons, RA and balloon-based strategies improved the mechanical performance of PCI in calcified lesions [16]. Taken together, the newer randomized evidence shows improvements most consistently for imaging and mechanical endpoints, whereas evidence of clinical event reduction remains limited and inconsistent, and the direct randomized comparisons between technologies remain few.

### 3.8. Intravascular Imaging Findings

Table 4 lists the imaging findings of studies whose primary or key endpoint was imaging-based. Entries are descriptive: the studies used different imaging modalities, endpoints and comparators, and were not pooled. In the randomized comparisons, no device consistently outperformed another for stent expansion or minimal stent area.

### 3.9. Risk of Bias in the Randomized Trials

Risk-of-bias judgments are shown in Table 5. Full-text reports sufficient for a domain-level judgment were available for five out of the eight randomized trials. ROLLER COASTR-EPIC22 was rated as high risk overall because final OCT, which provided the primary endpoint, was available in 135 out of 171 patients (79%), with no sensitivity analysis for the missing data; randomization used a web-based system before the procedure, the OCT analysis was performed by an independent core laboratory blinded to the treatment arm, the analysis was by intention to treat and the trial was registered. ROTA.shock, ROTA-CUT, ISAR-CALC and the Zou trial were rated as having some concerns overall. In ROTA.shock, allocation used envelopes (opaque or sealed status not stated), operators were not blinded, two patients randomized to lithotripsy crossed over to atherectomy, the primary analysis was restricted to patients with a final OCT (61 out of 70; nine missing, seven because of a software error and two because the catheter could not be advanced), the registration (6 August 2019) followed the start of enrollment, and the non-inferiority margin was derived from the observed atherectomy result. In ROTA-CUT, the method of sequence generation and allocation concealment was not described, the registered analysis could not be compared with the report, and alternative stent-expansion analyses were reported as performed but not presented. In ISAR-CALC, concealment was not described, additional non-compliant balloon dilation was used more often in the scoring-balloon group (32.4% versus 5.4%), OCT after stenting was available in 70 out of 74 patients (94.6%), and blinding of the OCT analysts was not explicitly stated. In the Zou trial, allocation used a random-number table “in order of interventional treatment”, which may mean quasi-sequential allocation (this domain was borderline high risk), no registration or prespecified primary outcome was reported, and outcome assessors were not described as blinded. No trial could blind operators to the device. For three further trials (ECLIPSE, EXIT-CALC and ORBID-FFR), only abstracts, registry records or secondary summaries were available, so ratings are provisional and most domains are not assessable: ECLIPSE and ORBID-FFR had some concerns in the domains that could be judged (ORBID-FFR also had a published sample size of 71 against 150 registered), and no domain could be judged for EXIT-CALC. Supporting statements for each judgment are listed in Supplementary Table S4.

### 3.10. Reporting Biases and Certainty of Evidence

Risk of bias due to missing results was not formally assessed (Section 2.7). One trial (ORBID-FFR) reported a sample size of 71 against 150 registered patients (Section 3.9). Certainty of evidence was not graded with GRADE or CINeMA. Because the evidence for each outcome came mainly from observational and small randomized studies, with risk-of-bias concerns, imprecise estimates and inconsistent outcome definitions, it was regarded as being of low certainty (Section 2.7).

## 4. Discussion

Procedural success was high with all calcium-modification strategies in the available comparative studies, and complications were generally uncommon. However, the evidence is heterogeneous in design, unit of analysis and endpoint definition, and success definitions differ between studies. The principal message of this review is therefore uncertainty rather than similarity: current evidence is insufficient to establish clinically meaningful differences between most calcium-modification technologies, and the absence of a reported difference should not be read as equivalence.

The evidence should be read in three tiers. Randomized head-to-head comparisons between technologies are few and small. Randomized comparisons against conventional balloon-based preparation (ECLIPSE and, after our search date, BALI, ShortCUT and VICTORY) test whether a technology adds value in a given lesion population and largely show gains in lesion preparation or stent expansion rather than reductions in clinical events. Observational comparisons make up most of the literature and are vulnerable to confounding by indication, because device choice follows lesion anatomy and operator preference. For example, in IVL-versus-RA comparisons, success was 99.3% vs. 95.8% in one retrospective study [38] and 89.4% vs. 89.1% in a registry comparing IVL with atherectomy of any type [31], with no significant differences. Likewise, ELCA, OA, and specialized balloons yielded success rates in the high 80s to 100% range. This is consistent with a recent meta-analysis reporting comparable clinical outcomes, including all-cause mortality, between IVL and RA [10]; that analysis also reported slight safety advantages for IVL (fewer dissections and shorter procedures), although it was derived mainly from observational data [10]. MACE definitions also varied substantially, which limits direct comparison and underscores the need for standardized MACE definitions in calcium-modification research.

Major complications were uncommon across interventions. Perforation ranged from 0% to 3.5% in the studies that reported it, in agreement with previous findings [10,11]. In IVL-versus-RA studies, perforation was below 1% in both groups and angiographic dissection was modest (0–1.3%) [38]. Slow or no reflow was 0–2% in most studies, although higher rates were reported in a few small studies (Section 3.3) [29,53,58]. These low event rates suggest acceptable safety profiles for modern calcium-modification techniques but are insufficient to exclude clinically relevant differences. The network meta-analysis of randomized trials by Maremmani et al. found a higher risk of serious adverse events with atherectomy-based strategies than with a conventional strategy, driven by slow flow/no reflow [15], a finding that our predominantly observational data can neither confirm nor refute. In-hospital or 30-day mortality and myocardial infarction were generally below 3% in the reported studies [33,34,38,39,44,45], although cardiac death at one year was 4.0% after OA and 2.7% after balloon angioplasty in ECLIPSE [27].

Longer-term outcomes were summarized descriptively and were not pooled; no quantitative synthesis of TLR, MACEs or mortality was performed. IVL-treated patients showed a trend toward better stent expansion in some studies [30,32,36], and a recent meta-analysis found higher rates of final stent deployment success and lower TLR in the IVL group [10], but these findings derive from pooled observational data and have wide confidence intervals. We therefore cannot conclude that any method reduces long-term events relative to another, nor that long-term efficacy is comparable.

ECLIPSE deserves emphasis. In 2005 patients with calcified lesions eligible for either strategy, routine OA did not reduce 1-year target-vessel failure (11.5% vs. 10.0%) and did not significantly increase minimal stent area compared with balloon angioplasty [27]. The lesson is not that OA and balloons are “equivalent”, but that when a lesion can be crossed and adequately prepared with balloon-based techniques, routine atherectomy did not improve the principal clinical or imaging outcomes. Success rates of 85% to 100% reported for OA in other studies [27] should be read in light of this.

The evidence base is evolving quickly. After our search date, BALI showed that adding IVL to conventional preparation reduced a composite dominated by imaging-defined residual stenosis [60]; ShortCUT and VICTORY suggested that cutting balloons and super-high-pressure balloons can be non-inferior to IVL for stent expansion in selected, crossable lesions [59,61]; and two broader randomized network meta-analyses reported more comprehensive quantitative comparisons than ours [15,16]. Together, these data indicate that the choice among technologies depends heavily on lesion phenotype and on the endpoint considered. An updated systematic search of multiple databases would be needed before any quantitative synthesis that includes these trials.

Relationship to existing network meta-analyses. The studies of Maremmani et al. [15] and Simonetti et al. [16] pooled randomized trials quantitatively and are broader than this review. Our review does not pool; its added value lies in the explicit separation of evidence tiers; the descriptive inclusion of OA, ELCA and observational data; the tabulation of imaging findings (Table 4); and the transparent statement of what the available evidence cannot support. Our conclusions regarding the limits of the evidence are consistent with theirs.

### 4.1. Imaging-Guided Selection of a Calcium-Modification Strategy

Contemporary practice is better described as a sequence of intravascular imaging, lesion phenotyping, strategy selection and imaging confirmation than as a choice among devices. IVUS and OCT can define calcium arc, thickness and length; nodular morphology; vessel dimensions; and balloon-crossability before treatment, and can verify calcium fracture, expansion and final minimal stent area afterwards; expert consensus recommends imaging for these purposes [4]. These findings can directly inform device selection, and minimal stent area and stent expansion are objective measures of procedural performance that are less susceptible to the definitional heterogeneity affecting “procedural success”. A recent review of intracoronary imaging in complex PCI likewise highlights its role in guiding strategy and identifying mechanisms related to procedural safety and outcomes [62]. Imaging likewise has a growing role in other complex lesion subsets, such as chronic total occlusions, where it supports lesion assessment, procedural planning and optimization [63], and imaging-guided PCI of calcified lesions has been examined in a recent network meta-analysis [64].

Imaging findings are summarized in Table 4. In the randomized comparisons, stent expansion was similar after IVL and conventional preparation [50], after IVL and RA (minimal stent area: 6.10 versus 6.60 mm^2^, within the prespecified non-inferiority margin of −1.60 mm^2^) [28], and after super-high-pressure and scoring balloons [51]. Observational imaging studies suggested better expansion with cutting or scoring balloons than with other balloons [55,57] and with OCT guidance than with IVUS guidance during RA [43]. Because imaging protocols, endpoints and lesion types differ, and several studies were small or single-arm, these findings are hypothesis-generating. Most studies did not report imaging-defined calcium morphology or stratify results by it, so imaging-defined subgroup analyses were not possible. This is not only a limitation but also a major reason why cross-study device comparisons may mislead, because calcium morphology is likely an important determinant of comparative device performance.

### 4.2. Lesion Phenotype and Anatomical Complexity

Device performance is likely to depend on lesion phenotype: superficial versus deep calcium, concentric versus eccentric distribution, calcified nodules, balloon-crossable versus uncrossable lesions, long calcified segments, chronic total occlusions, ostial and left main lesions, bifurcations, and under-expanded stents. These features act as effect modifiers, and differences in their distribution across studies limit any cross-study comparison and would threaten the transitivity assumption of an indirect comparison. Prior-CABG patients, for example, had worse outcomes that were attributed to more complex anatomy rather than to device performance [48].

Crossability deserves particular attention. Randomized comparisons usually enroll lesions that are eligible for several strategies; ECLIPSE, for instance, enrolled lesions eligible for both OA and balloon angioplasty [27]. Such trials under-represent balloon-uncrossable lesions, in which forward-cutting RA or ELCA may be the only option, and in which strategies that need balloon or crown delivery across the lesion (balloons; IVL; and, in part, OA) may not be usable or may need supportive maneuvers that carry their own risks. In crossable lesions, differences in success between devices may therefore be small and devices that avoid ablation may appear safer. Results from trials such as BALI, ShortCUT and VICTORY apply to selected, crossable, moderately to severely calcified lesions and should not be extrapolated automatically to the lesions in which atherectomy is most indispensable. We note this point as a clinical interpretation consistent with device mechanics rather than as a finding of our data.

### 4.3. Clinical Implications

Because randomized evidence cannot establish clinically meaningful differences between most technologies, device selection should be lesion-specific, imaging-informed, and based on deliverability, operator expertise and availability. For example, in balloon-crossable lesions, balloon-based strategies (including cutting or super-high-pressure balloons) or IVL may be considered, with IVL favored when deep or circumferential calcium is identified on imaging; RA or OA may be reserved for extremely rigid, nodular or balloon-uncrossable calcific lesions where ablative modification is beneficial. Selected studies suggest that specialty non-atherectomy balloons (for example, the Lacrosse NSE, a triple-scoring-element non-slip balloon) can be comparably effective to cutting balloons in moderately calcified lesions [49], but such data are limited. These insights align with expert consensus recommending intravascular imaging to guide therapy and acknowledging multiple viable calcium-modification modalities [4].

Selection of a calcium-modification device is only one component of the management of complex PCI. Severe calcification often coexists with other markers of procedural complexity, and post-PCI antithrombotic therapy should be individualized according to ischemic complexity and bleeding risk [65]. This does not imply that the use of atherectomy or IVL itself mandates more intensive antithrombotic treatment.

### 4.4. Limitations and Future Directions

This study has several limitations. The literature search was restricted to PubMed and ClinicalTrials.gov (with manual supplementation) and covered records published up to 1 April 2025; Embase, CENTRAL and other databases were not searched, so relevant studies may have been missed, and important randomized evidence has been published since. Most included comparisons were observational or single-center, introducing potential selection bias, confounding, and operator-driven treatment allocation. Study designs, outcome definitions, and follow-up durations varied, with some reporting in-hospital outcomes only, limiting comparability and precluding robust long-term inferences. Procedural success was defined differently across studies (for example, ECLIPSE used a more stringent definition and reported lower success than smaller studies), which limits comparison across studies and creates a ceiling effect. Study-level units of analysis differed (patients versus lesions), and multiple lesions per patient are not independent. Several randomized studies are sub-analyses of the same trial and were counted as one population. Risk of bias was formally assessed only for the randomized trials, certainty of evidence was not graded, the review was registered after the searches had been completed, and the methods departed from the registered protocol in several respects (Section 2.1 and Section 2.7), so conclusions should be read as low-certainty and hypothesis-generating. The bibliographic search used a single database (PubMed), filtered by publication type (Comparative Study or Randomized Controlled Trial), and so may have missed relevant studies that are not indexed under these types, including single-arm and observational reports.

The synthesis was descriptive. We did not pool effects, so we cannot estimate the size of any difference between devices or exclude a clinically relevant one. Only two independent randomized trials (ROTA.shock and ROLLER COASTR-EPIC22) were connected by a common comparator, and the remaining randomized trials compared devices with conventional balloons, tested adjuncts within one modality, or addressed imaging endpoints; a network meta-analysis would therefore have rested on a very small, unconnected evidence base. Short follow-up durations and low event rates further limited subgroup analyses and any formal consistency testing. Safety and clinical outcomes were summarized descriptively and were not pooled.

The overall quality and maturity of the evidence base remains a major limitation. Only a small number of randomized device-versus-device trials were available within the prespecified search period, most with modest sample sizes. The large ECLIPSE trial compared orbital atherectomy with modified balloon angioplasty rather than another atherectomy or lithotripsy device. Trials reported after the search period (BALI, ShortCUT, and VICTORY) are described narratively and are not part of the included set; they and other new trials may change the picture. Other randomized comparisons of atherectomy with balloon-based preparation (for example, ROTAXUS and PREPARE-CALC) address different questions. They are not among the included publications, which again reflects the single-database, publication-type-filtered search. Risk of bias could be rated from the full text for only five out of the eight randomized trials, none of which was at low risk overall; judgments for the other three are provisional, and observational studies were not formally rated with ROBINS-I; certainty of evidence was not graded.

Most studies did not stratify outcomes by calcification severity using standardized OCT or IVUS criteria, nor by anatomical complexity, precluding reliable subgroup analyses. This remains a key evidence gap, although emerging data suggest that non-atherectomy strategies may perform comparably in selected lesions.

Future research should prioritize adequately powered, head-to-head randomized trials directly comparing IVL, RA, OA, and other calcium-modification modalities, with standardized imaging-based definitions (including calcium morphology and balloon-crossability), longer follow-up, and harmonized clinical endpoints. Integration of cost-effectiveness analyses and evaluation of combination strategies (e.g., atherectomy followed by IVL or specialty balloons) will be essential to guide value-based, lesion-specific treatment selection in calcified coronary artery disease. An updated systematic search across multiple databases, with complete risk-of-bias assessment of all trials, ROBINS-I assessment of observational studies and formal certainty grading, is also needed.

## 5. Conclusions

Available comparative evidence indicates that contemporary coronary calcium-modification strategies generally achieve high procedural success with low complication rates, but the current evidence, which is largely observational and of low certainty, is insufficient to establish clinically meaningful differences among most technologies. This reflects imprecise and non-comparable data and does not demonstrate equivalence. Randomized trials more consistently show better lesion preparation or stent expansion than lower clinical event rates. Device selection should therefore remain lesion-specific and imaging-informed, incorporating calcium morphology, anatomical complexity, device deliverability, operator expertise, and local availability. Larger randomized head-to-head trials with standardized imaging definitions and harmonized clinical endpoints are required to define the relative roles of these technologies.

## Figures and Tables

**Table 1:** Procedural outcomes in randomized trials and comparative observational studies with ≥100 patients. The ROTA.shock OCT sub-studies derive from the same randomized population. Unit of analysis is patients unless stated.

Study	Comparison & Number (per Arm)	Procedural Success	Perforation	Dissection	Slow/No Reflow	Study Design	Unit of Analysis
ECLIPSE [27]	OA: 1 008 pts (1 250 lesions); BA: 997 pts (1 242 lesions)	OA 85.5%; BA 86.1% (per-protocol 84.0% vs. 82.8%)	n/r (very low)	n/r (very low)	OA 1.4%; BA 0.4%	RCT	Patients (clinical); lesions (procedural)
ROTA.shock [28,30]	RA: 37 pts; IVL: 33 pts (final OCT n = 61)	RA 100%; IVL 94%	0%	0% (angiography); OCT 77.8% (RA) vs. 75.0% (IVL)	0%	RCT	Patients
ROTA-CUT [37]	RA + CBA: 29 pts; RA + NCBA: 31 pts	RA + CBA 89.7%; RA + NCBA 96.8%	0% (both)	RA + CBA 21.4%; RA + NCBA 44.8%	0%	RCT	Patients
ROLLER COASTR-EPIC22 [36]	RA: 57 pts; IVL: 57 pts; ELCA: 57 pts	RA 93.0%; IVL 93.0%; ELCA 89.5%	RA 3.5%; ELCA 3.5%; IVL 0%	RA 1.7%; IVL 7.0%; ELCA 5.2%	RA 1.7%; IVL 0%; ELCA 1.7%	RCT	Patients
EXIT-CALC, Oomens [50]	IVL: 19 pts; conventional preparation: 21 pts	100% (all patients)	0%	IVL 63.2%; conv. 81.0% (*p* = 0.056)	n/r	RCT (single centre)	Patients
ISAR-CALC, Rheude [51]	SHPB: 37 pts; SB: 37 pts	SHPB 100%; SB 89.2% (*p* = 0.12)	2.7% (both)	SHPB 8.1%; SB 13.5%	n/r	RCT	Patients
Zou [29]	IVL: 20 pts; conventional PCI: 20 pts	100% (both)	0%	0%	5% (both)	RCT (single centre)	Patients
Kini, ORBID-FFR [58]	RA: 35 pts; CBA: 36 pts	n/r (primary endpoint: side-branch compromise 20.0% vs. 25.0%)	0%	RA 2.9%; CBA 0%	TIMI < 3: RA 8.6%; CBA 13.9%	RCT	Patients
Sukul [31]	IVL: 1 090 pts; any atherectomy: 1 743 pts	IVL 89.4%; atherectomy 89.1%	IVL 1.0%; atherectomy 2.1%	IVL 1.5%; atherectomy 1.2%	IVL 0.3%; atherectomy 1.4%	Observational (registry)	Patients
Zhao [38]	IVL: 152 pts; RA: 238 pts	IVL 99.3%; RA 95.8%	IVL 0.7%; RA 0.8%	IVL 0%; RA 1.3%	IVL 0%; RA 2.9%	Observational (retrospective)	Patients
Doshi [33]	OA: 91 pts; RA: 131 pts	TIMI 3 flow 100% (both)	0%	0%	n/r	Observational	Patients
Lee [34]	RA: 67 pts; OA: 50 pts	100% (both)	RA 0%; OA 2%	0%	RA 7%; OA 4%	Observational (retrospective)	Patients

Abbreviations: BA, balloon angioplasty; CBA, cutting balloon angioplasty; ELCA, excimer laser coronary angioplasty; IVL, intravascular lithotripsy; NCBA, non-compliant balloon angioplasty; OA, orbital atherectomy; OCT, optical coherence tomography; pts, patients; RA, rotational atherectomy; RCT, randomized controlled trial; SB, scoring balloon; SHPB, super high-pressure balloon; n/r, not reported. Success definitions differ between studies (Section 3.2), so rates are not directly comparable.

**Table 2:** Short- and medium-term clinical outcomes in randomized trials and comparative observational studies with ≥100 patients.

Study	Follow-Up	Restenosis	TLR	MACEs	Mortality	Study Design
ECLIPSE [27]	12 mo	n/r	n/r (target-vessel failure is shown under MACEs)	TVF: OA 11.5%; BA 10.0%	OA 4.0% CD; BA 2.7% CD	RCT
ROTA.shock [28]	1 & 6 mo	RA 0/33; IVL 1/28	RA 0/33; IVL 1/28 (restenosis)	None reported	0%	RCT
ROTA-CUT [37]	30 d	0%	0%	RA + CBA 10.3%; RA + NCBA 3.2%	0%	RCT
ROLLER COASTR-EPIC22 [36]	12 mo	n/r	Not yet reported	RA 0%; IVL 0%; ELCA 1.75%	0% (all arms)	RCT
EXIT-CALC [50]	1 mo	n/r	n/r	0% (both)	0% (both)	RCT
ISAR-CALC [51]	30 d	n/r	SHPB 0%; SB 8.1%	SHPB 0%; SB 10.8%	0% (both)	RCT
Zou [29]	6 mo	Stent stenosis ≥ 20%: IVL 10%; PCI 25% (not clinical restenosis)	0% (both)	0% (both; in-hospital & 6 mo)	0% (both)	RCT
Kini [58]	Not specified	n/r	n/r	n/r	None reported	RCT
Sukul [31]	In-hospital	n/r	n/r	IVL 4.3%; atherectomy 5.4%	IVL 2.7%; atherectomy 2.2%	Observational (registry)
Zhao [38]	In-hospital only	n/r	IVL 0%; RA 0.4%	IVL 0%; RA 0.8%	IVL 0%; RA 0.4%	Observational (retrospective)
Doshi [33]	In-hospital	n/r	n/r	MI: OA 2.9%; RA 6.4%	OA 0%; RA 1.5%	Observational
Lee [34]	30 d	n/r	0% (both)	6% (both)	RA 0%; OA 2%	Observational (retrospective)

Abbreviations: BA, balloon angioplasty; CBA, cutting balloon angioplasty; CD, cardiac death; ELCA, excimer laser coronary angioplasty; IVL, intravascular lithotripsy; MACEs, major adverse cardiovascular events; n/r, not reported; NCBA, non-compliant balloon angioplasty; OA, orbital atherectomy; RA, rotational atherectomy; TLR, target lesion revascularization; TVF, target-vessel failure.

**Table 3:** Summary of advantages and disadvantages of different calcium-modification techniques.

Technique	Advantages	Disadvantages
RA	Effective for concentric, dense, and nodular calcium Ablative calcium modification Can be used in lesions that balloons or other devices cannot cross Well studied with large real-world experience	Requires advanced operator expertise Higher risk of slow flow/no reflow compared with IVL or balloons Burr entrapment, wire bias, and thermal injury possible Higher cost than balloon-based techniques
OA	Treats both concentric and eccentric calcium Sanding mechanism allows bidirectional modification Can facilitate stent delivery in rigid lesions	Also requires specialized expertise Risk of slow flow/no reflow similar to RA Delivery requires crossing the lesion with the wire and crown Limited long-term outcome data Higher cost than balloons
IVL	Lower barotrauma compared with high-pressure balloons Effective for both superficial and deep calcium fractures Very low rates of perforation and slow flow Simple learning curve	High disposable cost Limited data in CTOs or very tight, uncrossable lesions Bulky balloon profile may limit crossing in severe stenosis
Cutting/Scoring Balloons	Controlled intimal scoring with lower inflation pressures Lower vessel trauma compared with high-pressure balloons Useful in focal calcium or fibrotic lesions	May be less effective in heavy circumferential calcium (historically); recent RCT data (ShortCUT, presented) show non-inferiority to IVL for minimal stent area in selected lesions Requires balloon crossing, so not suitable for very tight lesions Potential for dissections if calcium is deep or nodular
ELCA	Useful for non-balloon-crossable lesions to create a channel Effective for in-stent restenosis, under-expanded stents, and fibro-calcific plaque Can vaporize fibrocalcific material and thrombus	Requires operator expertise Less effective in heavy circumferential calcium compared with RA/OA/IVL Unique complications: thermal injury, dissection, vessel spasm, wire damage, microbubble-induced slow flow Higher cost than balloon-based techniques

Abbreviations: CTO, chronic total occlusion; ELCA, excimer laser coronary angioplasty; IVL, intravascular lithotripsy; OA, orbital atherectomy; RA, rotational atherectomy.

**Table 4:** Intravascular imaging findings in studies with an imaging endpoint.

Study (Ref.)	Design (n)	Modality	Comparison	Main Imaging Result	Comment
ROTA.shock [28,30]	RCT (n = 70; OCT 61)	OCT	IVL vs. RA	MSA 6.10 vs. 6.60 mm^2^ (difference: −0.50 mm^2^; non-inferiority margin: −1.60 mm^2^). Acute lumen gain: 0.17 vs. 0.46 mm^2^ (*p* = 0.03; sub-study n = 21)	Similar expansion; greater acute gain with RA; dissection: 75.0% vs. 77.8%
ROTA.shock nodules [35]	Sub-analysis of RCT (n = 19; RA 7, IVL 12)	OCT	RA vs. IVL	Neither modality significantly reduced calcified-nodule volume	Change attributed mainly to stent implantation; frequent eccentricity and malapposition
EXIT-CALC [50]	RCT (n = 40)	OCT	IVL vs. conventional preparation	Stent expansion: 83.9 ± 10.3% vs. 82.2 ± 11.5% (*p* = 0.63)	No difference
ISAR-CALC [51]	RCT (n = 74)	OCT	SHPB vs. scoring balloon	Stent expansion index: 0.72 ± 0.12 vs. 0.68 ± 0.13 (*p* = 0.22)	Better post-procedural lumen and less residual stenosis with SHPB
Kini [58]	RCT (n = 71)	OCT, FFR	RA vs. cutting balloon (side branch)	SB compromise: 20.0% vs. 25.0% (*p* = 0.62); SB FFR: 0.84 ± 0.08 vs. 0.83 ± 0.08	3D-OCT side-branch orifice area predicted SB FFR ≤ 0.80 (cut-off: 0.76 mm^2^; AUC: 0.92)
Kachi [54]	Retrospective (n = 38)	OCT	Scoring (n = 22) vs. cutting (n = 16) balloon after OA	Expansion ratio: 0.83 ± 0.20 vs. 0.68 ± 0.14 (*p* < 0.01); MLA: 3.95 vs. 3.43 mm^2^	Observational; allocation not randomized
Matsukawa [55]	Retrospective (n = 156)	IVUS or OFDI	Cutting balloon (n = 30) vs. NSE (n = 39) vs. Scoreflex (n = 87)	MSA: 7.78 ± 2.61 vs. 6.63 ± 2.15 vs. 6.83 ± 2.21 mm^2^; acute CSA gain: 5.89 vs. 4.38 vs. 4.60 mm^2^	Lower dilation pressure with the cutting balloon
Miyazaki [57]	Comparative (184 lesions; 130 pts)	IVUS	Scoring (n = 29) vs. conventional (n = 155) balloon before scaffold	Expansion index: 0.86 ± 0.20 vs. 0.71 ± 0.18 (*p* < 0.001); eccentricity index: 0.84 vs. 0.78 (*p* < 0.001)	Scoring group had more calcified lesions; TLR similar
Teng [43]	Retrospective (n = 79; 84 lesions)	OCT vs. IVUS guidance	OCT-guided vs. IVUS-guided RA	Stent expansion: 82 ± 8% vs. 75 ± 9% (*p* = 0.001)	Comparison of guidance, not of devices
Yamamoto [52]	Single-arm, pre/post OA (n = 29)	OCT	OA, before vs. after	MLA: 2.4 → 2.7 mm^2^ (*p* < 0.001); calcium area: 3.4 → 2.9 mm^2^ (*p* < 0.001)	Descriptive; no comparator

Abbreviations: CSA, cross-sectional area; IVL, intravascular lithotripsy; IVUS, intravascular ultrasound; MLA, minimal lumen area; MSA, minimal stent area; NSE, non-slip element balloon; OA, orbital atherectomy; OCT, optical coherence tomography; OFDI, optical frequency-domain imaging; RA, rotational atherectomy; RCT, randomized controlled trial; SB, side branch. Values are as reported in each study (mean ± SD, or median [IQR]).

**Table 5:** Risk of bias (RoB 2, effect of assignment) in the randomized trials.

Trial (Ref.)	D1	D2	D3	D4	D5	Overall	Basis
ECLIPSE [27]	Some concerns	Some concerns	Not assessable	Not assessable	Not assessable	Some concerns	Abstract and summaries only (provisional)
ROTA.shock [28,30,35]	Some concerns	Some concerns	Some concerns	Some concerns	Some concerns	Some concerns	Full text (PDF), main report
ROLLER COASTR-EPIC22 [36]	Low	Low	High	Low	Low	High	Full text (PDF)
ROTA-CUT [37]	Some concerns	Some concerns	Not assessable	Low	Some concerns	Some concerns	Publisher full text; registry and supplement not seen
EXIT-CALC [50]	Not assessable	Not assessable	Not assessable	Not assessable	Not assessable	Not assessable	Abstract and registry only
ISAR-CALC [51]	Some concerns	Some concerns	Some concerns	Some concerns	Some concerns	Some concerns	Publisher full text; registry not seen
Zou [29]	Some concerns	Some concerns	Low	Some concerns	Some concerns	Some concerns	Publisher full text
Kini, ORBID-FFR [58]	Some concerns	Not assessable	Not assessable	Not assessable	Some concerns	Some concerns	Abstract and protocol only (provisional)

Abbreviations: RoB 2, Cochrane Risk of Bias tool, version 2; D1, randomization process; D2, deviations from intended interventions; D3, missing outcome data; D4, measurement of the outcome; D5, selection of the reported result. “Not assessable”, the published text did not allow a judgment for that domain; “Not assessed”, no source describing the methods could be accessed and no rating was inferred; “provisional”, based on an abstract, registry record or secondary summary only. Judgments are based on published reports; registry records, protocols and statistical analysis plans were not consistently accessible.

## Data Availability

All data analyzed in this study were derived from published studies cited in the article. The extracted study-level data and risk-of-bias judgments are provided in Supplementary Tables S2–S4. The data extraction form is available from the corresponding author upon reasonable request. No analytic code was used, because no statistical pooling was performed.

## Supplementary Materials

The following supporting information can be downloaded at https://www.mdpi.com/article/10.3390/jcm15218319/s1: Table S1, PubMed search strategy; Table S2, Study-level audit of the 40 extracted studies: design, sample size, techniques compared, procedural success, evidence tier, overlap between publications and eligibility decision. Thirty-two publications were included and eight were excluded (four did not evaluate coronary calcium modification, two had bibliographic details that could not be verified at the time of the eligibility audit, and two had no usable outcome data); Table S3, Outcome data for the 32 included publications, as extracted (study-specific definitions); Table S4, RoB 2 judgments (effect of assignment) for the eight randomized trials, with the supporting statement for each domain. Five trials were rated from the full text; the ratings for ECLIPSE, EXIT-CALC and ORBID-FFR are provisional (abstract, registry record or secondary summary only). Observational studies were not rated with ROBINS-I; File S1, PRISMA 2020 checklist and PRISMA 2020 for Abstracts checklist. References [18,19,20,21,22,23,24,25,27,28,29,30,31,32,33,34,35,36,37,38,39,40,41,42,43,44,45,46,47,48,49,50,51,52,53,54,55,56,57,58] are cited in the Supplementary Materials.
