# Supplementary material for: Calcium-Modification Techniques in Calcified Coronary Artery Disease: A Systematic Review of Comparative Evidence on Procedural Success, Complications and Clinical Outcomes
Source: J Clin Med. 2026 Oct 26;15(21):8319. doi: 10.3390/jcm15218319 (PMC13764366; doi:10.3390/jcm15218319)
Supplement: Supplementary file 1 [file jcm-15-08319-s001.zip › Supplementary_File_S1_PRISMA2020_Checklist-edited.pdf]

Supplementary File S1. PRISMA 2020 Checklists

Manuscript jcm-4562911: "Calcium-Modification Techniques in Calcified Coronary Artery Disease: A Systematic Review of Comparative Evidence on Procedural Success, Complications and Clinical Outcomes."

Locations refer to section, table and figure numbers in the revised manuscript.

Table S5. PRISMA 2020 checklist

Section and topic	Item #	Checklist item	Location where item is reported
**TITLE**			
**Title**	1	Identify the report as a systematic review.	Title page (title)
**ABSTRACT**			
**Abstract**	2	See the PRISMA 2020 for Abstracts checklist.	Abstract; see Table 2 of this file
**INTRODUCTION**			
**Rationale**	3	Describe the rationale for the review in the context of existing knowledge.	Section 1 (Introduction), paragraphs 1–5
**Objectives**	4	Provide an explicit statement of the objective(s) or question(s) the review addresses.	Section 1, final paragraph; Section 2.2
**METHODS**			
**Eligibility criteria**	5	Specify the inclusion and exclusion criteria for the review and how studies were grouped for the syntheses.	Section 2.4, paragraph 2 (criteria); Section 2.6, "Grouping for synthesis"
**Information sources**	6	Specify all databases, registers, websites, organisations, reference lists and other sources searched or consulted to identify studies. Specify the date when each source was last searched or consulted.	Section 2.3, paragraphs 1–3 (searches run May 2025; targeted update April 2026); Abstract
**Search strategy**	7	Present the full search strategies for all databases, registers and websites, including any filters and limits used.	Supplementary Table S1 (PubMed); Section 2.3, paragraph 1 (ClinicalTrials.gov)
**Selection process**	8	Specify the methods used to decide whether a study met the inclusion criteria of the review, including how many reviewers screened each record and each report retrieved, whether they worked independently, and if applicable, details of automation tools used in the process.	Section 2.4, paragraph 1 (three independent reviewers); Section 2.3, paragraph 2 (no automation tools)
**Data collection process**	9	Specify the methods used to collect data from reports, including how many reviewers collected data from each report, whether they worked independently, any processes for obtaining or confirming data from study investigators, and if applicable, details of automation tools used in the process.	Section 2.4, data-extraction paragraph (two independent reviewers; investigators not contacted)
**Data items**	10a	List and define all outcomes for which data were sought. Specify whether all results that were compatible with each outcome domain in each study were sought (e.g. for all measures, time points, analyses), and if not, the methods used to decide which results to collect.	Section 2.5; Section 2.4, data-extraction paragraph; Supplementary Table S3 (study-specific definitions)

Section and topic	Item #	Checklist item	Location where item is reported
	10b	List and define all other variables for which data were sought (e.g. participant and intervention characteristics, funding sources). Describe any assumptions made about any missing or unclear information.	Section 2.4, data-extraction paragraph (variables; missing data recorded as n/r, no assumptions made)
**Study risk of bias assessment**	11	Specify the methods used to assess risk of bias in the included studies, including details of the tool(s) used, how many reviewers assessed each study and whether they worked independently, and if applicable, details of automation tools used in the process.	Section 2.7, paragraphs 1–2 (RoB 2; three independent reviewers)
**Effect measures**	12	Specify for each outcome the effect measure(s) (e.g. risk ratio, mean difference) used in the synthesis or presentation of results.	Section 2.6, "Effect measures"
**Synthesis methods**	13a	Describe the processes used to decide which studies were eligible for each synthesis (e.g. tabulating the study intervention characteristics and comparing against the planned groups for each synthesis (item #5)).	Section 2.6, "Grouping for synthesis"; Section 3.1, paragraph 2; Supplementary Table S2
	13b	Describe any methods required to prepare the data for presentation or synthesis, such as handling of missing summary statistics, or data conversions.	Section 2.6, "Effect measures" (no conversion, recalculation or imputation)
	13c	Describe any methods used to tabulate or visually display results of individual studies and syntheses.	Section 2.6, "Presentation of results and exploration of heterogeneity"; Tables 1, 2 and 4; Supplementary Table S3
	13d	Describe any methods used to synthesize results and provide a rationale for the choice(s). If meta-analysis was performed, describe the model(s), method(s) to identify the presence and extent of statistical heterogeneity, and software package(s) used.	Section 2.6, paragraph 1 (descriptive synthesis; rationale for not pooling); no meta-analysis performed
	13e	Describe any methods used to explore possible causes of heterogeneity among study results (e.g. subgroup analysis, meta-regression).	Section 2.6, "Presentation of results and exploration of heterogeneity" (descriptive only); Section 2.2
	13f	Describe any sensitivity analyses conducted to assess robustness of the synthesized results.	Section 2.6 (not performed, as no pooled estimates were produced)
**Reporting bias assessment**	14	Describe any methods used to assess risk of bias due to missing results in a synthesis (arising from reporting biases).	Section 2.7, final paragraph (not formally assessed; rationale given)
**Certainty assessment**	15	Describe any methods used to assess certainty (or confidence) in the body of evidence for an outcome.	Section 2.7, paragraph 1 (GRADE/CINeMA not applied; rationale given)
**RESULTS**			
**Study selection**	16a	Describe the results of the search and selection process, from the number of records identified in the search to the number of studies included in the review, ideally using a flow diagram.	Section 2.4, study-selection paragraphs; Figure 1 (PRISMA 2020 flow diagram)
	16b	Cite studies that might appear to meet the inclusion criteria, but which were excluded, and explain why they were excluded.	Section 2.4, study-selection paragraphs; Figure 1; Supplementary Table S2
**Study characteristics**	17	Cite each included study and present its characteristics.	Section 3.1; Table 1; Supplementary Table S3

Section and topic	Item #	Checklist item	Location where item is reported
**Risk of bias in studies**	18	Present assessments of risk of bias for each included study.	Section 3.9; Table 5; Supplementary Table S4 (randomized trials); Supplementary Table S2 (design features of observational studies)
**Results of individual studies**	19	For all outcomes, present, for each study: (a) summary statistics for each group (where appropriate) and (b) an effect estimate and its precision (e.g. confidence/credible interval), ideally using structured tables or plots.	Sections 3.2–3.5 and 3.8; Tables 1, 2 and 4; Supplementary Table S3 (group-level results; effect estimates where reported by the original studies)
**Results of syntheses**	20a	For each synthesis, briefly summarise the characteristics and risk of bias among contributing studies.	Sections 3.1 and 3.9; Tables 1 and 5
	20b	Present results of all statistical syntheses conducted. If meta-analysis was done, present for each the summary estimate and its precision (e.g. confidence/credible interval) and measures of statistical heterogeneity. If comparing groups, describe the direction of the effect.	Sections 3.2–3.5 and 3.8 (descriptive synthesis; no statistical synthesis performed)
	20c	Present results of all investigations of possible causes of heterogeneity among study results.	Sections 3.2–3.3 (definitions), 3.6 (subgroups) and 4.2
	20d	Present results of all sensitivity analyses conducted to assess the robustness of the synthesized results.	Not applicable (no sensitivity analyses; Section 2.6)
**Reporting biases**	21	Present assessments of risk of bias due to missing results (arising from reporting biases) for each synthesis assessed.	Section 3.10
**Certainty of evidence**	22	Present assessments of certainty (or confidence) in the body of evidence for each outcome assessed.	Section 3.10 (not formally graded; regarded as low certainty)
**DISCUSSION**			
**Discussion**	23a	Provide a general interpretation of the results in the context of other evidence.	Section 4, paragraphs 1–7
	23b	Discuss any limitations of the evidence included in the review.	Section 4.4, paragraphs 1–4
	23c	Discuss any limitations of the review processes used.	Section 4.4, paragraphs 1–2
	23d	Discuss implications of the results for practice, policy, and future research.	Sections 4.3 and 4.4 (final paragraph); Section 5
**OTHER INFORMATION**			
**Registration and protocol**	24a	Provide registration information for the review, including register name and registration number, or state that the review was not registered.	Section 2.1 (PROSPERO CRD420251077471, registered 25 June 2025); Abstract
	24b	Indicate where the review protocol can be accessed, or state that a protocol was not prepared.	Section 2.1 (full protocol available through the PROSPERO record)
	24c	Describe and explain any amendments to information provided at registration or in the protocol.	Section 2.1, “Amendments to the registered protocol” (timing of registration and seven changes from the protocol); Section 4.4

Section and topic	Item #	Checklist item	Location where item is reported
**Support**	25	Describe sources of financial or non-financial support for the review, and the role of the funders or sponsors in the review.	Funding statement (no external funding); Abstract
**Competing interests**	26	Declare any competing interests of review authors.	Conflicts of Interest statement
**Availability of data, code and other materials**	27	Report which of the following are publicly available and where they can be found: template data collection forms; data extracted from included studies; data used for all analyses; analytic code; any other materials used in the review.	Data Availability Statement; Supplementary Tables S2–S4

From: Page MJ, McKenzie JE, Bossuyt PM, et al. The PRISMA 2020 statement: an updated guideline for reporting systematic reviews. BMJ 2021;372:n71. doi:10.1136/bmj.n71. *This work is licensed under CC BY 4.0.*

Table S6. PRISMA 2020 for Abstracts checklist

Section and topic	Item #	Checklist item	Location where item is reported
**TITLE**			
**Title**	1	Identify the report as a systematic review.	Title
**BACKGROUND**			
**Objectives**	2	Provide an explicit statement of the main objective(s) or question(s) the review addresses.	Abstract, Background/Objectives
**METHODS**			
**Eligibility criteria**	3	Specify the inclusion and exclusion criteria for the review.	Abstract, Methods (sentence 1)
**Information sources**	4	Specify the information sources (e.g. databases, registers) used to identify studies and the date when each was last searched.	Abstract, Methods (sentence 2: searched May 2025)
**Risk of bias**	5	Specify the methods used to assess risk of bias in the included studies.	Abstract, Methods (final sentence)
**Synthesis of results**	6	Specify the methods used to present and synthesise results.	Abstract, Methods (descriptive synthesis without pooling)
**RESULTS**			
**Included studies**	7	Give the total number of included studies and participants and summarise relevant characteristics of studies.	Abstract, Methods (32 publications: 8 randomized trials, 22 observational) (total participants not summed because the unit of analysis differs between studies; sample sizes in Table 1)
**Synthesis of results**	8	Present results for main outcomes, preferably indicating the number of included studies and participants for each. If meta-analysis was done, report the summary estimate and confidence/credible interval. If comparing groups, indicate the direction of the effect (i.e. which group is favoured).	Abstract, Results
**DISCUSSION**			
**Limitations of evidence**	9	Provide a brief summary of the limitations of the evidence included in the review (e.g. study risk of bias, inconsistency and imprecision).	Abstract, Results (risk of bias) and Conclusions (observational, low certainty)
**Interpretation**	10	Provide a general interpretation of the results and important implications.	Abstract, Conclusions
**OTHER**			
**Funding**	11	Specify the primary source of funding for the review.	Abstract, Methods (no external funding)
**Registration**	12	Provide the register name and registration number.	Abstract, Methods (PROSPERO CRD420251077471)

From: Page MJ, et al. BMJ 2021;372:n71 (PRISMA 2020 for Abstracts). CC BY 4.0.
